# 

**Published:** 1894-06

**Authors:** 


WHOLE NUMBER, CCCXCIII.

New Series.

June, 1894.

VOL. XXXIII. No. 11.

LIBRARY
Indexed
S. G. O.

### BUFFALO MEDICAL AND SURGICAL JOURNAL.

Established 1845 by AUSTIN FLINT, M. D.

**Editors:**

THOS. LOTHROP, M. D. WM. WARREN POTTER, M. D.

**Associate Editors:**

WILLIAM C. KRAUSS, M. D. JAMES WRIGHT PUTNAM, M. D.

JOHN A. MILLER, PH. D. JOHN PARMENTER, M. D.

PUBLISHED MONTHLY.

SUBSCRIPTION PRICE, $2.00 IF PAID IN ADVANCE; OTHERWISE $2.50 A YEAR.

EUROPEAN AGENCY,
TRÜBNER & CO.
57 AND 59 LUDGATE HILL, LONDON, E. C.

BUFFALO:
1894.

FOREIGN
SUBSCRIPTION, $2.50

Entered at the Post-Office at Buffalo, N. Y., as Second-class Mail Matter.

*Times Printing House, Buffalo, N. Y.*

Table of Contents on Page V.

